# Pyroptosis in Skeleton Diseases: A Potential Therapeutic Target Based on Inflammatory Cell Death

**DOI:** 10.3390/ijms25169068

**Published:** 2024-08-21

**Authors:** Qian Wu, Jiacheng Du, Eun Ju Bae, Yunjung Choi

**Affiliations:** 1 Department of Biochemistry and Molecular Biology, Jeonbuk National University Medical School, Jeonju 54896, Republic of Koreadujc96@sina.com (J.D.); 2 School of Pharmacy, Jeonbuk National University, Jeonju 54896, Republic of Korea; 3 Division of Rheumatology, Department of Internal Medicine, Jeonbuk National University Medical School, Jeonju 54896, Republic of Korea; 4 Research Institute of Clinical Medicine of Jeonbuk National University-Biomedical Research Institute of Jeonbuk National University Hospital, Jeonju 54907, Republic of Korea

**Keywords:** pyroptosis, inflammation, skeleton diseases, treatment, cell death

## Abstract

Skeletal disorders, including fractures, osteoporosis, osteoarthritis, rheumatoid arthritis, and spinal degenerative conditions, along with associated spinal cord injuries, significantly impair daily life and impose a substantial burden. Many of these conditions are notably linked to inflammation, with some classified as inflammatory diseases. Pyroptosis, a newly recognized form of inflammatory cell death, is primarily triggered by inflammasomes and executed by caspases, leading to inflammation and cell death through gasdermin proteins. Emerging research underscores the pivotal role of pyroptosis in skeletal disorders. This review explores the pyroptosis signaling pathways and their involvement in skeletal diseases, the modulation of pyroptosis by other signals in these conditions, and the current evidence supporting the therapeutic potential of targeting pyroptosis in treating skeletal disorders, aiming to offer novel insights for their management.

## 1. Introduction

### 1.1. Pyroptosis: From Discovery to Development

Cell death is a fundamental physiological process that marks the cessation of critical cellular functions, including metabolism, growth, reproduction, response, and adaptability. Both insufficient and excessive cell death can result in pathological conditions, such as tumorigenesis and various acute injuries [1]. Regulated Cell Death (RCD) [2] is recognized as a gene-encoded mechanism present in multicellular organisms and some unicellular eukaryotes [3]. The primary role of RCD is to eliminate aging, damaged, and potentially harmful cells, thereby maintaining homeostasis. According to the Nomenclature Committee on Cell Death 2018 recommendations [4], RCD can be categorized into 12 distinct types, each involving complex procedures and contributing to various physiological and pathological processes.

In recent years, pyroptosis, a novel form of RCD, has garnered significant attention. In 1992, a self-destructive cellular phenomenon was first observed in *Shigella flexneri*-infected macrophages, bringing pyroptosis into focus [5]. In 1999, D. Hersh et al. reported apoptosis-like morphological changes in *Salmonella*-infected macrophages [6]. However, subsequent research revealed that the morphology of dead macrophages induced by *Salmonella* infection was distinct from that of apoptotic cells, particularly in the loss of an intact cell membrane [7,8]. Cookson et al. coined the term “pyroptosis,” derived from the Greek “pyro” (fire or fever) and “ptosis” (falling) [9]. In 2015, Shi et al. identified the pivotal role of gasdermin D (GSDMD) in pyroptosis, significantly advancing our understanding of this process [10]. Briefly, under stimuli such as reactive oxygen species (ROS), toxins, and pathogens, cellular inflammasomes assemble and activate caspase-1, which cleaves GSDMD, releasing its N-terminal domain (GSDMD-N) to form pores in the cell membrane. This leads to cell death as cellular contents are expelled [11]. Concurrently, caspases cleave the precursors of interleukin-1β (IL-1β) and interleukin-18 (IL-18), triggering their secretion and driving a potent inflammatory response [12]. Thus, pyroptosis is characterized as a pro-inflammatory programmed cell death [9].

Pyroptosis has been implicated in the development and progression of various inflammation-associated diseases, including cardiovascular disease [13], neurological disorders [14], liver diseases [15], and cancer [16,17]. Studies have suggested that modulating inflammation through pyroptosis—either by protecting normal cells from damage or by promoting the death of abnormal cells—offers a promising therapeutic approach. Skeletal disorders such as osteoarthritis (OA) [18], rheumatoid arthritis (RA) [19], and osteoporosis (OP) [20] are prevalent conditions that cause chronic pain, mobility impairments, and even disability, severely impacting the quality of life and imposing a substantial social burden [21]. Since inflammation and cell death are central to the pathology of these conditions, targeting pyroptosis presents a novel therapeutic strategy. Recent research highlights the growing interest in pyroptosis within the skeletal system. For instance, in knee osteoarthritis, increased NLRP3 inflammasome components and GSDMD levels were associated with heightened macrophage death, while inhibiting pyroptosis reduced synovitis in rats [22]. Another study demonstrated that inducing mitochondrial oxidative stress could trigger pyroptosis in osteosarcoma cells, enhancing the efficacy of anti-PD-L1 immunotherapy and significantly suppressing tumor growth [23]. Consequently, this review aims to elucidate the mechanism of pyroptosis, emphasize its significance in skeletal diseases, and explore its potential as a therapeutic target.

### 1.2. A Quick Look at Pyroptosis

Pyroptosis has been the subject of extensive exploration over the past several years, revealing both similarities and distinctions with other forms of RCD such as apoptosis and necroptosis (Table 1). One of the most noticeable features of pyroptosis is the dynamic morphological changes in affected cells. Activated gasdermin proteins bind to the membranes of compromised cells, forming oligomeric pores that allow the influx of external water, ultimately leading to cell swelling and rupture of the plasma membrane [24,25]. These pores also act as non-selective channels for the release of inflammatory factors and intracellular contents, contributing to the flattening of the cytoplasm [26,27]. In contrast, apoptotic cells retain intact plasma membranes, with the cytoplasm contracting rather than swelling. Additionally, membrane blebbing, a hallmark of apoptosis, also occurs in pyroptosis before cell rupture [28,29]. Pyroptotic bodies, which emerge from bubble-like protrusions, are similar in size to apoptotic bodies and can be observed under an electron microscope [27]. While pores also form in necroptotic cells, they are mediated by the MLKL pathway and are ion-selective [30]. The resultant change in osmotic pressure causes significant swelling, leading to an explosive cellular burst characteristic of necroptosis [31]. Further insights into pyroptosis can be gained by examining the nucleus and organelles. Unlike the fragmented nuclei seen in apoptosis, pyroptotic cells exhibit intact nuclei, though both cell death types display chromatin condensation [32,33]. Additionally, pyroptotic cells often show swollen mitochondria with reduced matrix density [34], a feature that distinguishes them from the specific mitochondrial alterations seen in apoptosis and necroptosis [35,36].

At the biochemical level, gasdermin proteins [37] and caspases [38] are key components of the pyroptosis pathway. Gasdermins serve as the executioners of pyroptosis, while caspases are essential for activating gasdermins. Gasdermins, except for DFNB59, consist of an N-terminal domain (gasdermin-N) and a C-terminal domain (gasdermin-C) [39]. The full-length gasdermins are cleaved by upstream effectors, removing the autoinhibitory gasdermin-C domain and allowing gasdermin-N to bind to the plasma membrane via membrane lipids, where it forms 10–14 nm pores [39]. The caspase family, an evolutionarily conserved group of intracellular cysteine endopeptidases [40], is categorized into three main types based on their cellular roles: modulating inflammation, participating in apoptosis, and influencing the cell cycle [41]. Caspases have become synonymous with cell death due to their involvement in these processes. Specifically, caspase-1, human caspase-4/5, and mouse homolog caspase-11 cleave GSDMD, mediating the canonical and non-canonical pathways of pyroptosis, respectively [10]. Caspase-3/6/7/8/9/10 (in humans) are key mediators of apoptosis [42]. Intriguingly, caspase-3 and caspase-8 have been found to induce pyroptosis by using GSDME and GSDMD as substrates, further underscoring the close relationship between pyroptosis and apoptosis [43,44].

Functionally, pyroptosis plays a critical role in adaptive immunity and inflammation. Initially discovered in the context of anti-infective immunity, pyroptosis is triggered to eliminate infected cells, preventing the spread of infection—a vital immune defense mechanism against pathogens. As research has progressed, the role of pyroptosis in tumor immunity has gained significant attention. Pyroptotic cells release immunologically active components that attract immune cells to tumor sites and activate tumor-specific immunity, highlighting the considerable anticancer potential of pyroptosis. However, pyroptosis can also facilitate tumor progression in certain contexts, reflecting its dual nature [17]. Due to its pivotal role in immune processes, pyroptosis is classified as a form of immunogenic cell death. The most prominent feature of pyroptosis is the intense inflammation it induces, which is evident throughout the process. This begins with the assembly of inflammasomes and requires pro-inflammatory caspases to activate gasdermins. Additionally, one of the outcomes of pyroptosis is the maturation and release of inflammatory cytokines IL-1β and IL-18. Nonetheless, it is important to note that the inflammatory response is not unique to pyroptosis; other mechanisms also lead to the maturation of IL-1 family members [45,46]. Finally, the dual-edged nature of pyroptosis must be acknowledged. While it can be beneficial in promoting pro-inflammatory cell death, excessive inflammation can result in damage to normal tissue structures. Therefore, only controlled activation of pyroptosis can exert a positive therapeutic effect.

**Table 1:** Comparison between pyroptosis, apoptosis, and necroptosis.

Cell Death Types	Morphological Characteristics			Key Components	Refs.
	Cell Membrane	Cytoplasm and Organelles	Nucleus		
Pyroptosis	Formation of membrane pores; membrane blebbing; formation of pyroptotic bodies; membrane rupture	Cytoplasmic swelling; swollen mitochondria with reduced matrix density	Intact nucleus; chromatin condensation	Caspase-1/3/4/5/8/11; gasdermins; inflammasomes	[24,25,27,32,34,37,38]
Apoptosis	Intact cell membrane; membrane blebbing; formation of apoptotic bodies	Reduced cellular volume; swollen mitochondria; MOMP	Nuclear fragmentation; chromatin condensation	Caspase-2/3/6/10; Bcl-2 family; death receptors; TNF receptor superfamily members	[28,33,35,38,47]
Necroptosis	Formation of membrane pores; membrane permeabilization and rupture	Cytoplasmic and organelle swelling	Dilated perinuclear space; sickle nucleus	MLKL; RIPK1; RIPK3	[30,31,36,48,49]

MOMP: mitochondrial outer membrane permeabilization; Bcl-2: B-cell lymphoma 2; TNF: tumor necrosis factor; and RIPK: receptor-interacting protein kinases.

## 2. Signaling Pathways of Pyroptosis and Their Significance in Skeleton Diseases

**Figure 1:**
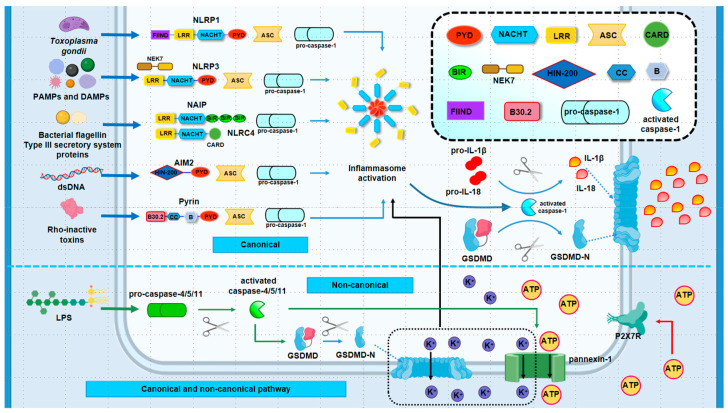
Mechanisms of canonical and non-canonical pyroptosis pathway. (1) Canonical pathway: In response to PAMPs and DAMPs, inflammasome sensors are activated, leading to the recruitment of ASC and pro-caspase-1 for inflammasome assembly. This results in the cleavage of pro-caspase-1 into activated caspase-1, which not only cleaves GSDMD to produce the cytotoxic GSDMD-N fragment but also cleaves pro-IL-1β and pro-IL-18 into their mature forms. GSDMD then forms pores in the cell membrane, mediating pyroptosis, while IL-1β and IL-18 are released through GSDMD-N pores to the extracellular environment; (2) non-canonical pathway: LPS directly activates pro-caspase-4/5/11, generating activated caspase-4/5/11, which cleaves GSDMD to form GSDMD-N, creating pores in the cell membrane. Additionally, K^+^ efflux through these pores activates the inflammasome, triggering the secretion of IL-1β and IL-18. Furthermore, caspase-4/5/11 can cleave pannexin-1, facilitating the release of ATP and K^+^, which in turn activates P2X7R to mediate pyroptosis. ASC: apoptosis-associated speck-like protein containing a caspase-recruitment domain; BIR: Baculovirus Inhibitor-of-apoptosis Repeat; CARD: caspase recruitment domain; FIIND: function-to-find domain; GSDMD: gasdermin D; IL-18: interleukin-18; IL-1β: interleukin-1β; LPS: lipopolysaccharide; LRR: leucine-rich repeats; NAIP: neuronal apoptosis inhibitory protein; NEK7: never in mitosis gene A (NIMA)-related kinase 7; and PYD: pyrin domain.

**Figure 2:**
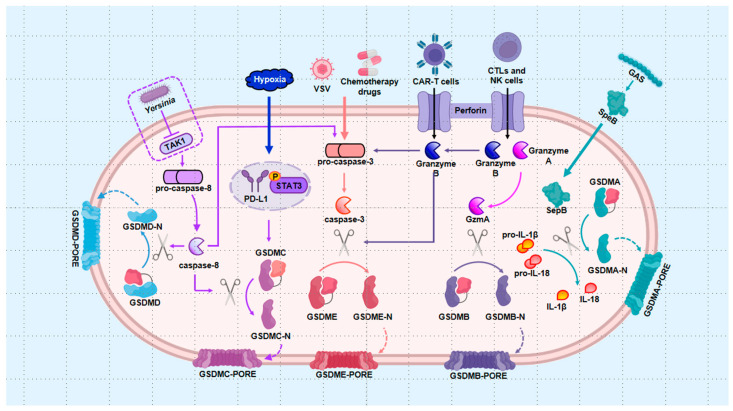
Alternative pathways. (1) Apoptotic caspases pathway: Chemotherapeutic drugs activate pro-caspase-3 into caspase-3, which subsequently cleaves GSDME, mediating pyroptosis in tumor cells. Yersinia inhibits TAK1, leading to the activation of caspase-8, which directly cleaves GSDMD and triggers caspase-3 activation, thereby inducing pyroptosis. Under hypoxic conditions, PD-L1 translocates into the nucleus with STAT3’s assistance, enhancing GSDMC expression and initiating GSDMC-N mediated pyroptosis. (2) Granzyme pathway: Granzyme B, released by CAR-T cells, CTLs, and NK cells, activates the caspase-3/GSDME axis, resulting in pyroptosis. Additionally, granzyme A directly cleaves GSDMB to form GSDMB-N pores; (3) GSDMA-mediated pathway: GAS-secreted SpeB cleaves GSDMA, leading to cell death. CAR-T: chimeric antigen receptor T-cell immunotherapy; CTLs: cytotoxic lymphocytes; GAS: Group A Streptococcus; GSDMA: gasdermin A; GSDMB: gasdermin B; GSDMC: gasdermin C; GSDMD: gasdermin D; GSDME: gasdermin E; IL-18: interleukin-18; IL-1β: interleukin-1β; NK cells: natural killer cells; SpeB: streptococcal pyrogenic exotoxin B; STAT3: signal transducer and activator of transcription 3; and TAK1: transforming growth factor (TGF) β-activated kinase 1.

Current research identifies two primary pathways for pyroptosis signal transduction: the pro-inflammatory pathway, encompassing both canonical and non-canonical routes (Figure 1), and several alternative pathways (Figure 2), including those mediated by apoptotic caspases, granzymes, and the recently discovered gasdermin A (GSDMA)-executed pathway. While these pathways converge on similar outcomes, they are initiated by distinct stimuli and require different effectors. The progression of pyroptosis hinges on gasdermin proteins forming pores in the cell membrane, leading to cell rupture and death, underscoring their role as the executors of pyroptosis. Among the five structurally similar gasdermins [50], GSDMD is particularly pivotal within the main pathway, whereas gasdermin E (GSDME), gasdermin C (GSDMC), and gasdermin B (GSDMB) are involved in alternative pathways. Recently, the GSDMA-mediated pyroptosis signaling pathway has also been identified. However, for gasdermins to exert cytotoxicity, they must first be processed to expose their functional N-terminal domains, which then insert into the plasma membrane. This cleavage is predominantly mediated by caspases, another critical component of pyroptosis. Caspase-1/4/5/11-driven pathways are classified as pro-inflammatory, while caspase-3/8-driven pathways are termed pro-apoptotic. The subsequent sections will detail the specific mechanisms of each key signaling pathway.

### 2.1. Pro-Inflammatory Pathway

#### 2.1.1. Canonical Inflammasome Pathway

##### A Quick Look at the Canonical Inflammasome Pathway

This pathway is orchestrated by caspase-1, initiated by a canonical inflammasome comprising three essential components. The first is the inflammasome sensor, also known as the pattern recognition receptor (PRR), which resides in the cytoplasm and includes the widely studied nucleotide-binding oligomerization domain (NOD)-like receptors (NLRs), absent in melanoma 2 (AIM2), and pyrin [51]. NLRs are composed of several key structures: the central NACHT domain, which contains the NOD, leucine-rich repeats (LRR), a pyrin domain (PYD), and a caspase recruitment domain (CARD) [51]. The NACHT domain is notable for its ATPase activity and its role in self-oligomerization [52], while the LRR primarily facilitates protein–protein interactions, contributing to ligand recognition and self-inhibition [53]. The PYD domain interacts with and aggregates apoptosis-associated speck-like protein containing a caspase-recruitment domain (ASC) through homotypic interaction [54], and the CARD domain is essential for recruiting pro-caspase-1 and inducing its activation [55]. NLRs can be further classified based on their N-terminal domains: NLRP1 and NLRP3, for example, both contain PYD and belong to the NLRP subgroup, with NLRP1 uniquely featuring a function-to-find domain (FIIND) absent in NLRP3. NLRC4, lacking PYD but possessing CARD, is classified under NLRCs, another NLR subgroup. Additionally, neuronal apoptosis inhibitory protein (NAIP), homologous to NLRC4, replaces CARD with three Baculovirus Inhibitor-of-apoptosis Repeats (BIRs) at the N-terminal [56]. NLRC4 activation requires binding to NAIP [56,57]. AIM2 and pyrin also have N-terminal PYD domains for ASC recruitment. AIM2, characterized by its HIN-200 domain at the C-terminal, acts as a sensor for bacterial or viral DNA, where the positively charged HIN-200 protein binds to the negatively charged dsDNA of pathogens, initiating host anti-infection immunity through electrostatic attraction [58,59,60,61]. Pyrin, on the other hand, detects bacterial toxins that inactivate Rho guanosine triphosphatase (Rho GTPase) [62]. Its C-terminal B30.2 domain is distinctive and directly interacts with caspase-1 [63]. The other two inflammasome components are ASC and pro-caspase-1, which have been described earlier. Understanding the composition of inflammasomes clarifies the assembly process. The initial step involves PRRs recognizing pathogen-associated molecular patterns (PAMPs) and danger-associated molecular patterns (DAMPs). NLRP1 is activated by *Toxoplasma gondii* [64] and *anthrax* lethal toxin [65], while NLRP3 responds to ATP, pathogenic microorganisms, their nucleic acids, and toxins [66]. The critical role of a specific protein in NLRP3 function warrants emphasis. The mitotic Ser/Thr kinase never in mitosis gene A (NIMA)-related kinase 7 (NEK7) facilitates the interaction with the LRR domain of NLRP3 upon its activation. This interaction is essential for the K^+^-efflux-dependent assembly and activation of NLRP3 inflammasomes [67,68]. This process can be negatively regulated by the centrosomal protein Spata2, which recruits the deubiquitinase CYLD to deubiquitinate polo-like kinase 4 (PLK4), leading to PLK4’s binding to phosphorylated NEK7 at Ser 204, thereby disrupting the interaction between NEK7 and NLRP3 [69]. NLRC4 activation is driven by bacterial flagellin and type III secretion system proteins, with NAIP’s assistance [70]. The stimuli for AIM2 and pyrin have already been discussed. Once activated, inflammasome sensors recruit pro-caspase-1 either directly via their own CARD (as seen with NLRC4) or through the ASC domain recruited by PYD, thereby completing inflammasome assembly. Activated caspase-1 then fulfills two critical roles: cleaving GSDMD to release its N-terminal (GSDMD-N), which induces pyroptosis, and processing pro-IL-1β and pro-IL-18 into their mature forms, which are secreted through GSDMD-N-created membrane pores [10,71]. This sequence culminates in cell pyroptosis as induced by caspase-1.

##### Canonical Inflammasome Pathway in Skeleton Disease

The canonical inflammasome pathway plays a pivotal role in host anti-infective immunity and is active in various cell types. For instance, *Staphylococcus aureus* (*S. aureus*) is a primary pathogen responsible for bone and joint infections. In studies with wild-type osteoblast-like MG-63 cells, *S. aureus* infection triggered inflammasome activation, leading to the release of caspase-1-dependent IL-1β. Additionally, caspase-1 restricted the proliferation of *S. aureus* in these cells, a response absent in *caspase-1*^−/−^MG-63 cells, where the bacterial load increased significantly. This suggests that osteoblasts, though non-professional phagocytes, contribute to host defense against *S. aureus* through caspase-1-mediated clearance [72]. However, as infection progresses, bone destruction becomes unavoidable, with NLRP3 contributing to osteoblast death and subsequent bone loss at the infection site [73]. Timely inhibition of caspase-1 and NLRP3 has been shown to reduce *S. aureus*-induced pyroptosis, thereby mitigating bone injury [74].

The components of this pathway are also implicated in other bone and joint diseases. The NLRP3/caspase-1/GSDMD axis is integral to maintaining the balance between osteogenesis and osteolysis, a process known as bone homeostasis. Osteoporosis, a systemic bone metabolic disorder, is characterized by the loss of trabecular bone volume and deterioration of bone microstructure, leading to an elevated risk of fragility fractures. Bone remodeling, the process by which osteoblasts generate new bone to replace old bone absorbed by osteoclasts, is crucial to preventing osteoporosis. An imbalance favoring osteoclast activity over osteoblast activity leads to bone mass loss [75]. It has been reported that activation of the NLRP3 inflammasome promotes adipogenic differentiation of mesenchymal stem cells (MSCs) while inhibiting osteogenic differentiation, a process reversible by caspase-1 inhibition [76]. A 2020 study found that oxidative stress induced by lipopolysaccharide (LPS) led to NLRP3-mediated pyroptosis in MG-63 cells, resulting in osteogenic disorders [77]. Following this, Tao et al. proposed that osteoblast pyroptosis could be a pathogenic mechanism underlying osteoporosis [78], a hypothesis that aligns with current findings. Additionally, IL-1β and IL-18, cytokines stimulated by NLRP3 activation, promote osteoclastogenesis [79]. The upregulation of IL-1β and IL-18 via the caspase-1 pathway exacerbates osteoblast death and osteoclast formation, further linking osteoporosis to pyroptosis. Paradoxically, the ASC domain has been shown to support osteoblast differentiation and osteogenesis, with slower bone defect repair observed in *Asc*-knockout mice compared to wild-type mice [80]. Moreover, *Nlrp3*-knockout mouse models have demonstrated NLRP3’s involvement in long bone induction and osteoblast maturation [81]. These insights suggest that NLRP3 has a complex and potentially contradictory role in bone formation and resorption, warranting further investigation.

Similar to osteoporosis, periodontitis is a disease characterized by bone resorption, leading to localized alveolar bone loss. Bioinformatics analysis has highlighted the involvement of pyroptosis-related genes in both periodontitis and osteoporosis, suggesting a potential link between these two conditions [82]. Shifting focus to periodontitis and its association with pyroptosis, studies using a periodontitis model induced by *Aggregatibacter actinomycetemcomitans* (Aa) demonstrated that caspase-1, rather than NLRP3, played a key role in promoting bone resorption [83], a finding that contrasts with earlier research [84]. These discrepancies may arise from differences in experimental models and the activation of alternative inflammasomes. Nevertheless, other reports have supported the notion that NLRP3 inflammasome activation enhances osteoclast differentiation and alveolar bone resorption [85,86]. According to recent research by Mohammad Ibtehaz Alam et al., NLRP3 regulates osteoclastogenesis in a bidirectional manner [87]. In the presence of LPS, NLRP3 accelerates osteoclast formation to remove pathogen-damaged bone tissue, indicating its positive role in the immune response. Conversely, NLRP3 negatively regulates osteoclast differentiation mediated by RANKL through pyroptosis, preventing excessive osteoclast activation and thereby maintaining bone homeostasis [87].

The inflammatory spread observed in periodontitis is also closely associated with various systemic diseases, including RA. RA is an autoimmune disease primarily characterized by chronic synovial inflammation, driven largely by immune cells within the synovial tissue [88]. During RA development, macrophages derived from peripheral blood monocytes differentiate into the proinflammatory M1 phenotype, releasing significant amounts of cytokines that contribute to severe inflammation [89]. Transcriptome analysis has suggested that pyroptosis-related genes, such as *AIM2* and *GPX4*, may be involved in the connection between periodontitis and RA [90]. This has drawn attention to the relationship between pyroptosis, inflammasomes, and RA. In RA models, macrophages exhibit significant increases in NLRP3-mediated caspase-1 activation, pyroptosis, and IL-1β release [91]. Additionally, elevated levels of GSDMD-N were detected in monocytes isolated from patients with RA [92], along with observable cell swelling and membrane blebbing under electron microscopy [92]. Monocytes incubated with serum from patients with RA displayed similar morphological changes [92], with upregulated expression of NLRP3, GSDMD-N, and IL-1β [92]. These results suggest that pyroptosis is active in RA and that the RA microenvironment directs monocytes to undergo NLRP3/GSDMD-mediated pyroptosis, leading to the secretion of inflammatory cytokines that further exacerbate joint destruction in RA [93]. Thus, pyroptosis is increasingly recognized as a potential pathogenic mechanism in RA.

Moreover, the expression of pyroptotic biomarkers is significantly upregulated in the synovium of patients with knee osteoarthritis (KOA) [94,95]. In fibroblast-like synoviocytes (FLSs), stimulation with LPS and ATP led to increased expression of NLRP1 and NLRP3 components, resulting in higher cell mortality compared to controls [94]. Silencing NLRP1 and NLRP3 via siRNA significantly inhibited LPS-induced inflammation and cell death, accompanied by a reduction in the protein and mRNA expression of pyroptosis markers ASC, caspase-1, and GSDMD [94]. Furthermore, inhibiting GSDMD in FLSs was shown to reduce synovial fibrosis, suggesting that targeting pyroptosis could mitigate synovial tissue damage [96]. Another study found that synovial macrophages, which are also implicated in KOA, undergo pyroptosis, as evidenced by their depletion in KOA rats [22]. Suppressing pyroptosis in these macrophages reduced synovial inflammation and fibrosis in KOA rats [22], providing further evidence that pyroptosis in synovial cells contributes to the pathogenesis of OA. Articular cartilage, the primary target tissue in OA, may also be affected by pyroptosis. While direct evidence is limited, cartilage degeneration driven by pyroptosis has been proposed as a possible mechanism in arthritis. Macrophages release inflammatory cytokines through pyroptosis, and DAMPs and PAMPs can directly activate pyroptosis in chondrocytes via the NLRP3 inflammasome, leading to IL-1β secretion. This, in turn, stimulates chondrocytes to produce catabolic enzymes that degrade cartilage [97,98]. Given cartilage’s critical role in bone growth and development in children, overactivation of the NLRP3/IL-1β axis in myeloid cells can lead to ischemia, hypoxia, impaired chondrocyte survival, and abnormal growth plate development [99].

Total joint replacement (TJR) remains the definitive treatment for severe joint degeneration. However, periprosthetic osteolysis (PPO) and subsequent graft aseptic loosening, often driven by wear particles, are significant postoperative complications. Wear particles stimulate surrounding cells to secrete pro-inflammatory mediators, which activate osteoclasts, promoting their differentiation, maturation, and inhibition of bone formation, ultimately leading to PPO [100]. It has been established that wear particles can induce osteocyte apoptosis [101], and osteocyte death has been shown to positively regulate osteoclastogenesis signals [102,103]. Given pyroptosis’s role as a pro-inflammatory form of cell death, it raises the question of whether pyroptosis underlies PPO. In a study where micro-sized tricalcium phosphate (TCP) particles were embedded in mouse calvaria to model osteolysis, increased intracellular ROS levels and heightened osteocyte oxidative stress were observed, activating NLRP3 inflammasomes in calvarial osteocytes [104]. Interestingly, inhibiting caspase-3-mediated apoptosis did not fully prevent osteocyte death, as the expression of caspase-1, GSDMD-N, and IL-1β increased, and the empty lacunae in osteocytes enlarged significantly. This confirmed that wear particles also induced pyroptotic death in osteocytes [104]. The inflammatory cytokines released during this process facilitated osteoclast formation, thereby promoting osteolysis [104]. These results suggest that pyroptosis plays a role in wear particle-mediated PPO. However, research on pyroptosis’s involvement in inducing PPO is still in its infancy, and further experimental validation is needed to confirm these preliminary conclusions.

Lumbar disc herniation (LDH) is a significant contributor to low back pain, primarily resulting from intervertebral disc (IVD) degeneration (IVDD). The IVD comprises the nucleus pulposus (NP), fibrous annulus (AF), and superior and inferior cartilage endplates (CEP). NP herniation not only mechanically compresses nerve roots but also acts as an immunologic stimulant, leading to nerve root inflammation and associated clinical symptoms. The NLRP3/caspase-1/IL-1β axis has been implicated in the pathogenesis of IVDD, which underlies LDH [105]. The prevailing view is that cell death and inflammation, predominantly within NP tissue, are central to the development of IVDD. *Propionibacterium acnes* (*P. acnes*) infection exacerbates disc degeneration [106]. He et al. demonstrated that when nucleus pulposus cells (NPCs) were co-cultured with *P. acnes*, the infected group exhibited elevated expression of NLRP3, along with upregulation of other pyroptosis-related proteins such as ASC and caspase-1, suggesting that *P. acnes* infection induces NPC pyroptosis via canonical inflammasome signaling pathways [107]. Subsequent research corroborated these findings, showing significant pyroptosis in human NPCs following co-culture with *P. acnes* [108]. In a rat model inoculated with *P. acnes*, there was a marked increase in IL-1β, IL-18, and caspase-1 levels, alongside evident IVD degeneration on MRI, indicating a substantial role for NPC pyroptosis in *P. acnes*-induced IVDD [108]. Additionally, elevated levels of NLRP3, caspase-1, and IL-1β were observed in patients with low back pain and Modic changes, a sign of cartilage endplate degeneration on MRI, further supporting the notion that the NLRP3/caspase-1/IL-1β axis is pivotal in promoting IVDD through CEP degeneration [109].

Vertebral structural degeneration or fractures can compress the spinal cord, leading to severe motor and sensory dysfunction. Traumatic spinal cord injury (SCI), often resulting from significant trauma, is a common cause of disability and death, and current treatments remain unsatisfactory. SCI progresses through two phases: the initial trauma causes primary mechanical injury to tissues and cells, followed by the release of highly pro-inflammatory contents from necrotic cells, which activate PRRs and trigger intense neuroinflammation [110]. Given the significant roles of cell death and inflammation in SCI, it is plausible that pyroptosis contributes to its pathogenesis. Dai et al. observed that the expression levels of NLRP3, ASC, caspase-1, GSDMD, and the concentrations of IL-1β and IL-18 were significantly higher in the SCI group compared to the sham group [111]. Zhang and colleagues reported similar findings in a mouse model of SCI [112]. Moreover, researchers noted damaged neurons exhibiting shrunken nuclei and extensive vacuolar degeneration in an SCI model induced by ischemia-reperfusion injury [113]. Elevated levels of dsDNA in serum and cerebrospinal fluid activated AIM2 inflammasomes, which in turn increased the expression of ASC, caspase-1, and IL-1β, indicating that neuronal pyroptosis plays a role in the progression of SCI [113].

#### 2.1.2. Non-Canonical Inflammasome Pathway

In the non-canonical pyroptosis pathway, inflammatory caspases—specifically human caspase-4/5 (caspase-11 in mice)—are activated in response to pathogen stimulation without the need for inflammasome sensors. Cytoplasmic LPS directly binds to the CARD domain of caspase-4/11, initiating caspase activation through oligomer formation [114]. Once activated, caspase-4/5/11 can cleave the pyroptosis executor protein GSDMD, producing a P30 fragment at the N-terminal that inserts into the cell membrane, leading to cytotoxic effects [10,115,116]. For instance, Brucella LPS activates caspase-11-mediated pyroptosis, which helps limit joint infection by killing macrophages and restricting bacterial survival [117].

Unlike caspase-1, caspase-4/5/11 does not directly process pro-IL-1β and pro-IL-18. Inhibition of caspase-5 does not reduce IL-1β levels [107]. The question then arises: how does the non-canonical pathway trigger inflammation? Researchers have found that the caspase-11/GSDMD pathway diverges at GSDMD-N, which not only transmits pyroptosis signals but also acts upstream to activate caspase-1 in the presence of NLRP3 and ASC [115,118]. GSDMD-N disrupts cell membrane integrity, and K^+^ efflux through the damaged membrane facilitates caspase-4/5/11 activation of NLRP3, leading to the secretion of mature IL-1β and IL-18 and an ensuing an inflammatory response [119]. Chen and colleagues demonstrated that *P. gingivalis* triggers pyroptotic death in periodontal ligament stem cells via a caspase-4-dependent non-canonical pathway [120]. In this process, GSDMD cleaved by caspase-4 mediates IL-1β release, inhibiting osteogenic differentiation and promoting osteoclast differentiation [120].

Furthermore, LPS stimulation prompts caspase-11 to interact with and cleave the pannexin-1 channel, facilitating ATP export, which then activates the P2X7 receptor (P2X7R) channel, inducing pyroptosis [121]. Pannexin-1 processed by caspase-11 also mediates K^+^ efflux, thereby activating NLRP3 and initiating IL-1β release [121]. Notably, a 2021 study revealed that elevated extracellular ATP in chondrocytes of OA rats activated P2X7R, leading to extracellular matrix degradation and pyroptotic inflammation via NF-κB/NLRP3 crosstalk, exacerbating OA progression, including increased cartilage damage and bone resorption [122]. These findings underscore the role of P2X7R-related pyroptosis in the development and progression of OA.

Additionally, caspase-4 has emerged as a potential therapeutic target for SCI based on bioinformatics analysis [123]. In vivo experiments confirmed increased caspase-4 expression in injured spinal cords, implicating caspase-4-dependent non-canonical pyroptosis in SCI pathology [123]. Inhibition of caspase-4 was shown to reduce inflammatory marker levels and improve injury outcomes, highlighting its therapeutic potential [123].

### 2.2. Alternative Pathways

In summary, exploring alternative pathways has significantly advanced our understanding of pyroptosis mechanisms. Notably, pyroptotic cell death can occur independently of inflammasomes. GSDME, a key member of the pyroptosis executor family, plays a pivotal role in these alternative pathways, alongside other gasdermin family members such as GSDMD, GSDMC, GSDMB, and GSDMA. The initiation of pyroptosis by pro-apoptotic caspase-3/8 underscores the complex interplay between different forms of cell death, highlighting the intricate relationships among them. Additionally, the identification of the granzyme pathway and the GSDMA-mediated pathway has further expanded our understanding of pyroptosis. These developments align with the revised definition of pyroptosis as a form of RCD that relies on gasdermin family proteins to form plasma membrane pores, a process often—but not exclusively—triggered by the activation of inflammatory caspases. However, alternative pathway-mediated pyroptosis remains underexplored in the context of bone diseases. With continued research, we anticipate that new and unexpected findings will emerge, further illuminating this field.

#### 2.2.1. Apoptotic Caspases Pathway

Caspase-3 was traditionally understood to regulate apoptotic signaling exclusively. However, GSDME, which is located downstream of caspase-3 and initially identified as DFNA5 [124], has redefined this understanding. Rogers et al. discovered that infection with the vesicular stomatitis virus led to the cleavage of the DFNA5 protein by caspase-3, generating a necrotic N-terminal structure that targeted the cell membrane and induced secondary necrosis [125]. Later that year, a pivotal study published in Nature confirmed that high expression of GSDME shifts caspase-3-activated cells from apoptosis to pyroptosis [43]. Chemotherapeutic drugs can activate caspase-3 in cancer cells, triggering GSDME-N-dependent pyroptosis [43], a process that also occurs in normal cells, thereby enhancing chemotherapy toxicity [43]. Subsequent research further revealed that GSDME plays a role in the release of IL-1β and IL-18, independently of pyroptotic cell death [126,127]. Today, the conversion of apoptotic signals to pyroptosis has been widely recognized as an effective mechanism in cancer treatment [128,129]. For instance, dioscin, a natural compound, has been shown to kill osteosarcoma cells and inhibit tumor growth through the caspase-3/GSDME axis-mediated pyroptosis [130]. Beyond its anti-tumor effects, abundant tumor necrosis factor-α (TNF-α) in the RA synovial environment has been found to elevate the expression of cleaved caspase-3 and GSDME-N, leading to synovial cell pyroptosis and promoting the secretion of inflammatory factors, thereby exacerbating arthritis severity [131,132].

Caspase-8, another apoptosis initiator, can also trigger pyroptosis, using GSDMD [44,133], GSDME [133], and GSDMC [134] as substrates. During *Yersinia* infection and subsequent transforming growth factor (TGF) β-activated kinase 1 (TAK1) inhibition, caspase-8 directly cleaves GSDMD or indirectly activates GSDME through caspase-3-mediated cleavage, and both pathways produce an active P30 fragment that leads to macrophage pyroptotic death [44,133]. The mechanism of inflammatory cytokine release in the caspase-8-mediated pyroptosis pathway is still debated, likely due to differences in experimental design. Orning et al. proposed that active GSDMD controls K^+^ efflux, which directs the activation of NLRP3 and subsequent secretion of IL-1β [44]. Conversely, Chen and colleagues suggested that pannexin-1 enables downstream NLRP3 to participate in IL-1β release [135]. Given that TAK1 is necessary for pro-IL-1β production, Sarhan et al. explained that caspase-8-activated inflammasomes in TAK1-inhibited cells might transfer to TAK1-active cells, where abundant pro-IL-1β promotes IL-1β secretion [133].

Additionally, under hypoxic conditions, PD-L1 is promoted to translocate to the nucleus with the assistance of the phosphorylated signal transducer and activator of transcription 3 (STAT3), enhancing GSDMC expression [134]. Caspase-8 then activates GSDMC, converting apoptosis to pyroptosis in cancer cells [134], a process that may also be initiated by certain antibiotic chemotherapeutic agents [134]. Interestingly, lumbar spinal stenosis has been associated with GSDMC expression in the Chinese population [136], though the specific role of GSDMC-dependent pyroptosis in lumbar spinal stenosis remains unclear.

#### 2.2.2. Granzyme Pathway

Three studies published in 2020 highlighted the role of granzymes in triggering pyroptosis. First, in the context of chimeric antigen receptor T-cell (CAR-T) immunotherapy, granzyme B released by CAR-T cells was shown to activate the caspase-3/GSDME axis in tumor cells, inducing pyroptosis [137]. Second, cytotoxic lymphocytes (CTLs) and natural killer (NK) cells were found to release granzyme B, which can directly cleave GSDME after Asp270, similarly to caspase-3, thereby driving pyroptosis in target cells [138]. Third, granzyme A released by CTLs was observed to enter cancer cells and cleave GSDMB at the Lys229/Lys244 site, generating GSDMB-N, which targets the plasma membrane to mediate pyroptosis [139].

#### 2.2.3. GSDMA-Mediated Pathway

Recently, researchers have drawn inspiration from the discovery that the cysteine protease streptococcal pyrogenic exotoxin B (SpeB), released by Group A *Streptococcus* (GAS), can directly cleave IL-1β [140]. This led to the finding that SpeB can also cleave GSDMA in a caspase-independent manner at the Gln246 site, generating GSDMA-N, which then inserts into and damages cell membranes, thereby causing pyroptosis [141,142]. In this straightforward signal transduction process, GSDMA proteins serve as critical junctures, responding to toxin stimulation and targeting cell membranes to initiate pyroptosis upon activation.

## 3. The Regulation of Pyroptosis in Cells with Bone Diseases

### 3.1. Macrophages

In bone diseases, macrophages play an important role in the inflammatory response by releasing pro-inflammatory cytokines. When macrophages undergo pyroptosis, these cytokines are released in large quantities, leading to increased local inflammation and thus affecting bone health. The role of the NLRP3 inflammasome in this process is central, as its activation not only promotes the release of pro-inflammatory cytokines like IL-1β and IL-18 but also leads to the cleavage of gasdermin D (GSDMD), a key executor of pyroptosis. NF-κB, a pivotal transcription factor in inflammatory signaling, further amplifies this process by upregulating NLRP3 and GSDMD, thus creating a feed-forward loop that exacerbates inflammation and tissue damage. Research by Fan and colleagues revealed that LPS-induced bone marrow-derived macrophages (BMDMs) exhibited mitochondrial damage, as indicated by a decrease in membrane potential. However, enhancing mitophagy, a selective form of autophagy, was found to suppress the activation of the NLRP3 inflammasome [143]. This suppressive effect was partially reversed by the mitophagy inhibitor 3-methyladenine (3-MA) [143]. Similar findings were reported by Hsieh et al. [144], suggesting that promoting autophagy could reduce macrophage pyroptosis and subsequently alleviate joint inflammation. NF-κB/RELA also contributes to pyroptosis in RA by upregulating NLRP3 and downregulating miRNA-30a, which normally suppresses NLRP3 expression by binding to its 3′-untranslated region (UTR) in macrophages [145]. Additionally, NF-κB enhances GSDMD transcription by binding to specific regions upstream of the GSDMD promoter [146]. Therefore, enhancing autophagy or inhibiting NF-κB in macrophages can reduce GSDMD-mediated pyroptosis.

### 3.2. Chondrocytes

Chondrocytes are key cells that maintain the structure and function of articular cartilage, and pyroptosis plays an important role in degenerative bone diseases such as osteoarthritis. When chondrocytes undergo pyroptosis, the activation of inflammasomes triggers the release of inflammatory cytokines, which further aggravate the degradation of cartilage matrix and lead to the destruction of cartilage tissue. In addition, the formation of cell membrane pores and the release of cell contents caused by pyroptosis lead to a sustained inflammatory response in the surrounding cartilage tissue, accelerating the degradation of articular cartilage. A previous study on chondrocytes corroborated these findings and revealed that Licochalcone A (Lico A) treatment upregulated Nrf2 and HO-1 expression while suppressing LPS-induced NLRP3 inflammasomes [147]. However, the protective effects of Lico A against pyroptosis were reversed by Nrf2-siRNA [147]. This study also highlighted that Lico A inhibited the NLRP3 inflammasome by blocking NF-κB and proposed that this inhibition was mediated through Nrf2’s role in preventing NF-κB nuclear translocation, a process supported by earlier evidence of Nrf2’s involvement in NF-κB modulation [147,148]. In OA cartilage, the number of caspase-1 and NLRP3-positive cells is significantly higher than in normal cartilage, coinciding with increased IκBα phosphorylation and p65 translocation to the nucleus [149,150]. Blocking IκBα phosphorylation and p65 nuclear translocation has been shown to inhibit pyroptosis [149], whereas administration of recombinant NF-κB restores pyroptosis levels [149]. These insights suggest that chondrocyte pyroptosis is positively regulated by upstream NF-κB signaling, which accelerates OA progression. Inflammation is also linked to the purinergic receptor P2X7R, which mediates Na^+^ and Ca^2+^ influx and K^+^ efflux following ATP stimulation. K^+^ efflux can trigger inflammation-related events, including activation of the NF-κB signaling pathway and NLRP3 inflammasomes [151]. Thus, P2X7R not only activates NF-κB signaling, which contributes to cartilage degradation via MMP-13 and increases NLRP3 levels, but also directly activates NLRP3 inflammasomes, leading to chondrocyte pyroptosis. This suggests that P2X7R exacerbates OA through the interplay between NF-κB and NLRP3, driving both pyroptosis and chondrocyte degradation [122]. Similarly, Toll-like receptor 4 (TLR4), a PRR, senses LPS and transmits the signal to downstream NF-κB. TLR4-mediated NF-κB signaling promotes the generation of NLRP3 and pro-IL-1β, while also activating NLRP3, leading to pyroptotic inflammation in RA macrophages [152]. Moreover, pyroptosis resulting from DNA polymerase beta (Pol β) deficiency is a newly identified mechanism in RA pathogenesis [153]. Pol β deficiency amplifies nuclear translocation of p65 and accumulates DNA damage, activating the cGAS-STING pathway, which further enhances NF-κB signaling and induces macrophage pyroptosis [153]. Interventions targeting these pathways have shown promise in mitigating pyroptosis and protecting cartilage.

### 3.3. Synovial Cells

Synovial cells are key cells that make up the synovium of joints and are usually responsible for maintaining the balance of fluid in the joint cavity and the normal function of the joint. However, in an inflammatory environment, synovial cells may undergo pyroptosis, a process that leads to the massive release of inflammatory factors in synovial cells through the activation of NLRP3 inflammasomes. The release of these inflammatory factors not only triggers an inflammatory response in the local synovium, but also leads to the proliferation and destruction of synovial tissue, further promoting pathological changes in the joints. In addition, the lysis of synovial cells and the leakage of contents caused by pyroptosis further stimulate the overreaction of the immune system, leading to persistent inflammation and destructive remodeling of the joint tissue. Restoring lysosomal function enhances autophagic flux, which leads to pain relief and cartilage protection. This improvement is associated with a reduction in the metabolic factor matrix metalloproteinase 13 (MMP-13) and IL-1β within the synovial tissue [154]. Rapamycin, an mTOR inhibitor, has been shown to prevent articular cartilage degradation by reducing MMP-13 levels in chondrocytes [154]. Given that MMP-13 expression is influenced by cytokines such as IL-1β, it is plausible to propose that autophagy-mediated reduction of pyroptosis and IL-1β secretion protects chondrocytes from MMP-13-induced damage. A recent study further supported this notion by demonstrating that rapamycin activated autophagy, which in turn downregulated MMP-13 expression and decreased the levels of the NLRP3 pyroptosis pathway components (NLRP3, caspase-1, GSDMD, and IL-1β), thereby mitigating OA progression by inhibiting chondrocyte pyroptosis [155]. Additionally, stromal cell-derived factor-1 (SDF-1) has been reported to inhibit NLRP3-mediated pyroptosis in OA-FLS, partially through the autophagy pathway [95]. Metabolic byproducts decrease synovial fluid pH, activating ASIC1a and promoting pyroptosis in chondrocytes [156]. Another possible mechanism is that joint inflammation activates NF-κB signaling, which increases ASIC expression, subsequently triggering pyroptosis [157]. Notably, the ASIC1a inhibitor amiloride has been shown to alleviate pyroptosis [156]. The increased intracellular Ca^2+^ concentration mediated by ASIC1a is a critical factor, as limiting intracellular Ca^2+^ levels have been found to reduce the severity of pyroptosis [156]. Further insights into ASIC1a’s regulation of pyroptosis reveal that Ca^2+^ activates calpains, which relieve the endogenous inhibition of calcineurin, leading to calcium-mediated cell death [158]. Inhibition of calcineurin has also been shown to downregulate NLRP3, ASC, and caspase-1 [159], suggesting that ASIC1a accelerates pyroptosis through the Ca^2+^/calpain/calcineurin axis. Additionally, pyroptosis triggered by ASICs has been observed in RA synoviocytes [160,161]. While direct evidence linking pyroptosis to this process is still lacking, it represents a promising avenue for future research.

### 3.4. Nucleus Pulposus Cell

The modulation of pyroptosis by autophagy is also evident in NPCs, where pyroptosis plays a significant role in IVDD [107,108]. Research has shown that autophagy activation in ROS-induced NPCs, achieved through the use of the autophagy activator rapamycin, results in increased expression of LC3II and decreased levels of cleaved caspase-1, IL-1β, and IL-18 [162]. Conversely, inhibiting autophagy significantly increased the expression of NLRP3, cleaved GSDMD, and cleaved caspase-1 [162,163]. These findings underscore the negative regulatory role of autophagy in pyroptosis, with the autophagy-lysosomal pathway playing a pivotal role in the degradation of the pyroptosis executor GSDMD-N [164]. When lysosomal function is impaired, the accumulation of GSDMD-N can accelerate pyroptosis in NPCs [164]. Mitochondrial damage in NPCs, induced by various stimuli, leads to ROS production and subsequent cellular inflammation and death [165,166,167]. Mitochondrial damage activates NLRP3 inflammasomes, resulting in elevated levels of NLRP3 protein and IL-1β [168]. Additionally, leakage of mitochondrial DNA (mtDNA) into the cytoplasm through the mitochondrial permeability transition pore (mPTP) can act as a DAMP, inducing pyroptotic cell death [169]. Maintaining mitochondrial function by reducing damage can inhibit the NLRP3 inflammasome signaling pathway and delay IVDD progression [168,169,170]. Mitophagy plays a critical role in maintaining mitochondrial homeostasis and has garnered significant attention from researchers due to its involvement in key pathways related to damage repair, inflammation, and pyroptosis. ROS, particularly mitochondrial ROS (mtROS), are key activators of the NLRP3 inflammasome [171]. As the predominant form of cellular ROS, mtROS plays a central role in these processes. The promotion of mitophagy by Sirtuin 1 (SIRT1) has been shown to reduce mtROS, suppressing NLRP3-mediated pyroptosis and providing protection against IVDD by preserving NPCs [172]. The regulatory role of Nrf2 in pyroptosis extends to IVDD and SCI as well. As with the aforementioned conditions, Nrf2 overexpression was found to reduce LPS/ATP-induced pyroptosis [173], while inhibition or knockdown of Nrf2 restored pyroptosis levels [162,174]. Notably, Nrf2 was shown to bind to the promoter region of microRNA (miRNA)-146a, enhancing its expression [173]. miRNA-146a, in turn, targets GSDMD, reducing its expression [173]. As expected, knocking down miRNA-146a negated Nrf2’s inhibitory effect on pyroptosis [173]. This indicates that Nrf2 suppresses pyroptosis by promoting miRNA-146a expression, thereby enhancing GSDMD inhibition. Therefore, targeting these pathways may provide therapeutic opportunities for delaying or preventing the progression of IVDD and other degenerative diseases associated with pyroptosis.

## 4. Pyroptosis as an Intervention Target to Treat Skeleton Diseases

### 4.1. Exosomes and miRNAs

Exosomes are extracellular vesicles with a diameter ranging from 40 to 160 nm (average 100 nm) that can be released by all cells and carry a variety of molecules, including cytokines, DNA, RNA, lipids, and proteins [175]. Functionally, exosomes modulate intracellular signal transduction and can be engineered to deliver therapeutic payloads to specific targets, offering significant therapeutic potential across various diseases [175]. Exosomes derived from MSCs contain elevated levels of miRNA-410, which can directly bind to the 3′UTR of NLRP3, inhibiting its expression and thereby preventing LPS-induced pyroptosis, ultimately improving IVDD [176]. Exosomes from human umbilical cord MSCs (HUCMSCs) deliver miRNA-26a-5p to reduce METTL14 expression, thereby curbing subsequent pyroptosis and enhancing NPC activity [177]. Building on the concept that exosomes can ameliorate IVDD by inhibiting pyroptosis, an exosome-coupled extracellular matrix hydrogel has been developed as a tissue engineering treatment for IVDD [178].

MiRNAs, small non-coding RNAs, play critical roles in post-transcriptional regulation and are involved in processes such as cell proliferation, differentiation, and death [179]. MiRNA-223-3p targets NLRP3 and inhibits MSU-induced pyroptosis in FLSs, suggesting its potential as a biomarker for GA treatment [180]. MiRNA-140-5p reduces the transcription of cathepsin B (CTSB), impairing the interaction between CTSB and NLRP3, thus repressing pyroptosis in chondrocytes [181]. MiRNA-219a-5p reduces FBXO3 expression, and its loss attenuates NLRP3-mediated pyroptosis in cartilage tissue [182]. MiRNAs can act upstream or downstream of the NLRP3 inflammasome to prevent pyroptosis. For instance, miRNA-326 restricts NF-κB signaling to protect chondrocytes from pyroptosis [183], while miRNA-107 targets caspase-1, downregulating its expression to block pyroptosis in chondrocytes [184]. MiRNA-204 not only reduces Ca^2+^ and ROS levels but also specifically binds to and inhibits GSDMD, thereby blocking pyroptosis in FLSs of AS. Notably, BMSC-derived exosomes delivering miRNA-326 have been reported to improve OA [183]. Therefore, exosomes delivering other therapeutic miRNAs could be explored for treatment, while excluding miRNA-155, miRNA-144-3p, and miRNA-30b-5p due to their adverse effects.

### 4.2. Non-Drug Therapy

Recent research has expanded the understanding of how SFA can intensify inflammation and reduce chondrocyte viability by enhancing the classical pyroptosis pathway [185]. In contrast, n-3 polyunsaturated fatty acids (PUFA), but not n-6 PUFA, have been shown to attenuate upstream TLR4/NF-κB signaling, thereby repressing the NLRP3/caspase-1 axis, combating inflammation, and protecting chondrocytes against pyroptosis [185]. The anti-pyroptosis effect of n-3 PUFA has been recognized in previous studies as well. For instance, maresin 1, an anti-inflammatory and pro-resolving mediator synthesized by macrophages from docosahexaenoic acid (DHA), a constituent of n-3 PUFA, alleviates inflammatory radicular pain by inhibiting pyroptosis through the NF-κB pathway [186]. In summary, a diet rich in n-3 PUFA is recommended to prevent and treat obesity-related OA due to its anti-inflammatory and anti-pyroptosis properties.

From a novel OA mechanism perspective, moderate-intensity exercise helps maintain P2X7R at an optimal level, thereby enhancing chondrocyte autophagy via the AMPK/mTOR pathway to alleviate pyroptosis [187]. Irisin, a myokine secreted during physical exercise that stimulates muscle contraction, has been reported to exert anti-OA effects in chondrocytes [188]. Jia et al. observed a reduction in Irisin levels within damaged OA cartilage. However, exercise, particularly of moderate intensity, markedly enhanced Irisin expression, stimulated the production of chondrocyte-specific collagen II, and diminished the number of MMP-13s, a disintegrin and metalloprotease with thrombospondin motifs 5 (ADAMTS-5), ADAMTS-5, NLRP3, and caspase-1 positive cells [150]. Further research confirmed that Irisin could suppress pyroptosis in chondrocytes by inhibiting the PI3K/Akt/NF-κB pathway [150]. Another anti-inflammatory molecule, Lipoxin A4 (LXA4), is produced in the capillary and infrapatellar fat pad during exercise. LXA4 inhibits NF-κB signaling and resists chondrocyte pyroptosis by promoting M2 polarization of synovial macrophages during moderate exercise [189].

### 4.3. Drug Therapy

#### 4.3.1. Melatonin

Obesity, a global public health concern, contributes to or exacerbates cardiovascular diseases, diabetes, and OA. Research has demonstrated that inflammation in adipose tissue plays a pivotal role in the development of chronic diseases [190]. Liu et al. showed that melatonin administration significantly reduced inflammasome marker levels in adipose tissue, suppressing inflammasome activation [146]. Additionally, melatonin reversed LPS-induced pyroptosis in adipocytes by inhibiting the accumulation and nuclear translocation of NF-κB/p65 [146]. The most crucial function of melatonin is its antioxidant capacity, primarily mediated through the activation of the Nrf2 pathway. Melatonin treatment enhances Nrf2 translocation to the nucleus, thereby inhibiting ROS-mediated activation of NLRP3 inflammasomes. Nrf2 knockdown completely blocked melatonin’s ability to mitigate inflammatory damage, underscoring the necessity of Nrf2 in melatonin’s anti-inflammatory effects [191]. In IVDD, melatonin not only restrains the harmful positive feedback loop of the IL-1β/NF-κB/NLRP3 axis in NPCs but also likely reduces mtROS production through Nrf2 activation [192]. Nicotinamide phosphotransferase (NAMPT) exacerbates IVDD progression by activating the NLRP3 inflammasome via mitogen-activated protein kinase (MAPK) and NF-κB pathways in NPCs. Melatonin, however, inhibits NAMPT expression, thereby alleviating matrix degradation [193].

#### 4.3.2. Common Drugs

Non-steroidal anti-inflammatory drugs (NSAIDs) are classic treatments for skeletal diseases, with inhibition of pyroptosis being one of their mechanisms. Indomethacin, a widely used NSAID, has been shown to decrease pyroptosis-related markers such as caspase-1, IL-1β, and IL-18 at both mRNA and protein levels in OA chondrocytes. Interestingly, this anti-inflammatory effect is enhanced by GANT-61, an inhibitor of the downstream gene GLI in the Hedgehog signaling pathway [194]. Ipriflavone, another therapeutic agent used for treating OP, regulates the activity of osteoblasts and osteoclasts. Chen et al. demonstrated that Ipriflavone inhibits mtROS-induced NLRP3 inflammasome activation in macrophages, thereby facilitating early bone healing [195]. However, it is worth noting that bisphosphonates, a different class of anti-OP drugs, can mediate their side effect of osteonecrosis through activation of the canonical NLRP3/caspase-1/IL-1β pathway [196]. Diabetes is known to increase the risk of skeletal diseases [197]. Glyburide, a medication for treating type 2 diabetes mellitus (T2DM), also acts as an NLRP3 inhibitor, reducing the expression of pro-inflammatory cytokines and decreasing the number of osteoclasts in diabetes-induced fractures, while simultaneously promoting endochondral bone formation and mineralization, thereby accelerating fracture healing [198]. Metformin, a prominent drug in diabetes management, has been shown to block dsDNA/AIM2-mediated pyroptosis in macrophages associated with diabetes [199]. Beyond its role in diabetes therapy, metformin also mitigates ischemia-reperfusion injury by dampening cardiomyocyte pyroptosis through the AMPK/NLRP3 pathway [200]. The anti-pyroptosis effect of metformin has also been utilized in the treatment of skeletal diseases, as it reduces NLRP3-mediated pyroptosis in cartilage and the spinal cord, thereby protecting chondrocytes and neurons, offering new therapeutic avenues for OA and SCI [201,202].

## 5. Conclusions and Prospects

Pyroptosis, a form of RCD intricately linked to inflammation, has rapidly become a focal point in research, with new mechanisms and regulatory pathways continuously emerging. This form of cell death is both beneficial and detrimental. Initially identified in macrophages infected by *Shigella flexneri* and *Salmonella*, pyroptosis is recognized for its positive role in anti-infection immunity. Additionally, there is growing interest in leveraging pyroptosis to induce tumor cell death, potentially restricting tumor growth, metastasis, and recurrence. However, more often than not, pyroptosis acts as a pathological agent, exacerbating cell death under disease conditions and provoking intense local inflammation, which can lead to significant tissue damage.

Skeletal diseases, which cause pain, mobility impairments, and disability, have a profound impact on patients’ quality of life. In severe cases, such as with fractures and malignant bone tumors, these conditions can even be life-threatening. Research into the role of pyroptosis in skeletal diseases has advanced rapidly, with numerous studies confirming its key role in the onset and progression of these conditions. Similar to its dual nature in other diseases, pyroptosis can sometimes serve as an ally, aiding in the defense against bone and joint infections and inhibiting osteosarcoma proliferation. However, pyroptosis more often acts as an adversary, contributing to the pathogenesis of various forms of arthritis, OP, and IVDD. In arthritis, pyroptosis in chondrocytes and synoviocytes accelerates disease progression. The resulting accumulation of pro-inflammatory cytokines inhibits osteoblast differentiation while excessively activating osteoclasts, driving the development of OP. Furthermore, the inflammatory death of NPCs hastens the degeneration of intervertebral discs. Given these roles, pyroptosis presents itself as a potential therapeutic target for skeletal diseases. Inhibiting key components of pyroptosis, such as inflammasomes, caspases, and gasdermins, could help alleviate symptoms and slow disease progression.

In conclusion, this review provides an overview of the signaling pathways and regulatory mechanisms of pyroptosis, highlighting their relevance in skeletal diseases. It also compiles current evidence supporting the treatment of skeletal diseases through the inhibition of pyroptosis. Looking forward, the goal is to translate the therapeutic potential of targeting pyroptosis into effective treatments, making the use of pyroptosis inhibitors a standard approach for managing inflammatory skeletal diseases.

## Abbreviations

3-MA: 3-methyladenine; Aa: *Aggregatibacter actinomycetemcomitans*; ADAMTS-5: a disintegrin and metalloprotease with thrombospondin motifs 5; AF: fibrous annulus; AIM2: absent in melanoma 2; AMPK: Adenosine 5′-monophosphate (AMP)-activated protein kinase; AS: ankylosing spondylitis; ASC: apoptosis-associated speck-like protein containing a caspase-recruitment domain; ASICs: Acid-sensing ion channels; BA: Baicalein; BET: bromodomain and extra-terminal domain; BIRs: Baculovirus Inhibitor-of-apoptosis Repeats; BMDMs: bone marrow-derived macrophages; BRD4: Bromodomain-containing protein 4; CARD: caspase recruitment domain; CAR-T: chimeric antigen receptor T-cell immunotherapy; CEP: cartilage endplate; CTLs: cytotoxic lymphocytes; CTSB: cathepsin B; DAMPs: danger-associated molecular patterns; DEPTOR: DEP domain containing mTOR interacting protein; DHA: docosahexaenoic acid; DUBs: Deubiquitinases; FBXL2: F-box L2; FBXO3: F-box O3; FIIND: function-to-find domain; FLSs: fibroblast-like synoviocytes; FOXO3a: forkhead box O3; GA: gouty arthritis; GAS: Group A *Streptococcus*; GSDMA: gasdermin A; GSDMB: gasdermin B; GSDMC: gasdermin C; GSDMD: gasdermin D; GSDME: gasdermin E; HDAC: histone deacetylase; HIF-1α: hypoxia-inducible factor-1α; HO-1: Heme Oxygenase-1; HUCMSCs: human umbilical cord MSCs; IGF2BPs: insulin-like growth factor 2 mRNA-binding proteins; IKK: IκB kinase; IL-18: interleukin-18; IL-1β: interleukin-1β; IRF: interferon regulatory factor; IVD: intervertebral disc; IVDD: intervertebral disc degeneration; KOA: knee OA; LC3B: light chain 3B; LDH: Lumbar disc herniation; Lico A: Licochalcone A; LPS: lipopolysaccharide; LRR: leucine-rich repeats; LXA4: Lipoxin A4; m^6^A: N^6^-methyladenosine; MAPK: mitogen-activated protein kinase; miRNA: microRNA; MMP-13: metalloproteinase 13; mPTP: mitochondrial permeability transition pore; MSCs: mesenchymal stem cells; MSU: Monosodium urate; mtDNA: mitochondrial DNA; mTOR: mechanistic target of rapamycin; mtROS: mitochondrial ROS; NAIP: neuronal apoptosis inhibitory protein; NAMPT: Nicotinamide phosphotransferase; NEK7: never in mitosis gene A (NIMA) -related kinase 7; NF-κB: nuclear factor kappa-B; NK cells: natural killer cells; NLRs: nucleotide-binding oligomerization domain (NOD) -like receptors; NOX: NAD(P)H oxidases; NP: nucleus pulposus; NPCs: nucleus pulposus cells; Nrf2: Nuclear factor-erythroid 2-related factor 2; NSAIDs: Non-steroidal anti-inflammatory drugs; OA: osteoarthritis; OP: osteoporosis; OTU: ovarian tumour domain containing proteases; *P. acnes*: *Propionibacterium acne*; P2X7R: P2X7 receptor; PAMPs: pathogen-associated molecular patterns; PEMF: Pulsed electromagnetic field; PI3K: phosphoinositide-3 kinase; PINK1: putative kinase 1; PLK4: polo-like kinase 4; Pol β: polymerase beta; PPO: periprosthetic osteolysis; PRR: pattern recognition receptor; PTEN: phosphatase and tensin homolog; PUFA: polyunsaturated fatty acids; PYD: pyrin domain; RA: rheumatoid arthritis; RANKL: receptor activator for nuclear factor-κB ligand; RCD: Regulated Cell Death; Rho GTPase: Rho guanosine triphosphatase; ROS: reactive oxygen species; *S. aureus*: *Staphylococcus aureus*; SCFAs: short-chain fatty acids; SCI: spinal cord injury; SCIRI: spinal cord ischemia-reperfusion injury; SDF-1: stromal cell-derived factor-1; SFA: saturated fatty acids; SIRT1: Sirtuin 1; SMAD2: SMAD family member 2; SpeB: streptococcal pyrogenic exotoxin B; STAT3: signal transducer and activator of transcription 3; T2DM: type 2 diabetes mellitus; TAK1: transforming growth factor (TGF) β-activated kinase 1; TCP: tricalcium phosphate; TJR: total joint replacement; TLR4: Toll-like receptor 4; TNFAIP3: TNF-α induced protein 3; TNF-α: tumor necrosis factor-α; TXNIP: Thioredoxin-interacting protein; USP: ubiquitin-specific protease; UTR: untranslated region; and ZnF: zinc-finger.
